# Effects of Synthetic Astaxanthin on the Growth Performance, Pigmentation, Antioxidant Capacity, and Immune Response in Black Tiger Prawn (*Penaeus monodon*)

**DOI:** 10.1155/2023/6632067

**Published:** 2023-12-22

**Authors:** Qiang Chen, Shuting Huang, Jieyu Dai, Congcong Wang, Songming Chen, Yuanxin Qian, Yangyang Gong, Tao Han

**Affiliations:** ^1^Department of Aquaculture, Zhejiang Ocean University, Zhoushan 316000, China; ^2^Zhejiang NHU Co., Ltd., Shaoxing 312500, China

## Abstract

Synthetic astaxanthin is an effective nutritional strategy for improving shrimp body color and promoting growth. However, the optimal amount of astaxanthin in feed also varies with the synthetic technology and purity. In the present study, five diets containing different doses of synthetic astaxanthin (0% (CON), 0.02% (AX0.02), 0.04% (AX0.04), 0.08% (AX0.08), and 0.16% (AX0.16)) were administered to *Penaeus monodon* (initial body weight: 0.3 ± 0.03 g) for 8 weeks. With an increase in astaxanthin content in feed, weight gain and specific growth rate increased initially and subsequently decreased, with the highest value appearing at AX0.08. Dietary astaxanthin supplementation obviously improved the carapace and muscle color by enhancing astaxanthin pigmentation. Meanwhile, the fatty acid profile was altered by dietary astaxanthin, as evidenced by a decline in palmitic acid proportion, along with an increase in n-3 polyunsaturated fatty acids (n-3 PUFA) contents in muscle. In addition, dietary astaxanthin supplementation regulated prawn's antioxidant capacity. In the hemolymph, the activities of glutamic pyruvic transaminase (GPT) showed a significantly decrease trend with linear effect. The activities of glutamic oxaloacetic transaminase (GOT) and the contents of malondialdehyde (MDA) were first downregulated and then upregulated with significantly quadratic pattern. In the hepatopancreas, the activities of superoxide dismutase (SOD) and the contents of MDA were significantly downregulated with the increase of dietary astaxanthin levels. Reduced glutathione (GSH) contents and catalase (CAT) activities were also significantly decreased in group AX0.08. Correspondingly, astaxanthin decreased GSH and MDA contents under transportation stress. Moreover, the mRNA expression of immune genes (*traf6*, *relish*, and *myd88*) were inhibited by dietary astaxanthin supplementation. Based on the results of polynomial contrasts analysis and Duncan's test, dietary synthetic astaxanthin is a suitable feed additive to improve the growth, body color, antioxidant capacity, and nonspecific immunity of *P. monodon*. According to the second-order polynomial regression analysis based on the weight gain, the optimal supplementation level of dietary astaxanthin was 90 mg kg^−1^ in *P. monodon*.

## 1. Introduction

Black tiger prawn (*Penaeus monodon*), together with Chinese white shrimp (*Fenneropenaeus chinensis*) and Whiteleg shrimp (*Litopenaeus vannamei*), is among the largest shrimp species globally and is famous for its fast growth rate, delicious flavor, and high-nutritive value [1]. In the past decade, the overall production of *P. monodon* has shown an upward trend in China [2]. As a commercial aquaculture species, coloration attracts consumers and plays a vital role in price [3]. However, *P. monodon* under intensive culture conditions with artificial compound feed is poor colorful compared with prawns in the wild environment.

Dietary pigments supplementation seems to be an appropriate option for improving color and increasing commercial value of shrimp [4]. Carotenoids, also known as tetraterpenoids, are widely used as pigments in shrimp due to their benefits in improving growth and enhancing body color [5]. Common carotenoid pigments include astaxanthin, *β*-carotene, and canthaxanthin [6]. Among them, astaxanthin is proved to be the most predominant pigment in shrimp [7]. In addition to improve body color, astaxanthin also promotes shrimp health through enhancing the immune response [8] and relieving oxidative stress [9]. Astaxanthin is the strongest natural antioxidant to date and activates the Keap1-Nrf2 system to scavenge free radical produced from external stress [10, 11]. Moreover, astaxanthin is involved in the process of metabolism regulation. In mammals, studies have indicated that astaxanthin relieves lipid deposition by promoting fatty acid oxidation and suppressing fatty acid *de novo* synthesis [12]. Fatty acid profiles were also influenced by astaxanthin [13]. Hence, other than its role as a pigment and antioxidant, astaxanthin also has a significant regulatory effect on the lipid metabolism.

Although astaxanthin has many significant functions, shrimp cannot synthesize astaxanthin on their own and mainly absorb astaxanthin through diets [14]. In general, natural astaxanthin used in aquaculture is extracted from *Haematococcus pluvialis* [15]. While chemically synthetic astaxanthin is also widely used in the feed of shrimp because of its price and purity advantage over natural astaxanthin [16]. During the past years, there are many studies on astaxanthin in shrimp, including comparing of synthetic astaxanthin and natural astaxanthin [17], improving body color [18] and enhancing antioxidant ability [19]. However, astaxanthin with different chemical structure and isomerism have different effects on shrimp [20]. Hence, the optimum amount of astaxanthin in shrimp feed also varies with the source of astaxanthin. The synthetic astaxanthin in the present study has been proved to be better than natural astaxanthin in previous study of our laboratory [21]. Correspondingly, five diets containing different level synthetic astaxanthin were fed *P. monodon* for 8 weeks aiming to evaluate the optimization dietary astaxanthin of juvenile *P. monodon*. The results are expected to provide theoretical reference for the optimization of feed formulation in *P. monodon*.

## 2. Materials and Methods

### 2.1. Experimental Diets Preparation

Five experimental diets containing different astaxanthin levels (the purity of synthetic astaxanthin was 10% and was synthesized by Zhejiang NHU Co., Ltd., Shaoxing, China) with equal crude protein (46.85%) and lipid (11.02%) contents were created. Diet AX0 (control diet), containing no astaxanthin, served as the foundation for the current trial (Table 1). Whereas, the other four diets were supplemented with 0.02% (20 mg kg^−1^), 0.04% (40 mg kg^−1^), 0.08% (80 mg kg^−1^), and 0.16% (160 mg kg^−1^) astaxanthin (diets AX0.02, AX0.04, AX0.08, and AX0.16). The process of feed preparation included crushing, mixing, pelleting, and drying. Finally, the pellets were kept at −20°C until usage after being dried at 55°C for 12 hr in a ventilated oven.

### 2.2. Prawns, Experimental Conditions, and Procedure

*P. monodon* juveniles were obtained from Zhongzheng Aquatic Science and Technology Co., Ltd., Hainan, China. Experimental prawns were fed in tanks for 14 days to acclimate the diet and environment. Healthy prawns with similar weight (initial body weight: 0.3 ± 0.03 g) were fasted for 24 hr and randomly divided into 15 tanks (300 L), 25 prawns per tank. Prawns were fed five times at 5:00, 10:00, 15:00, 19:00, and 23:00 every day for 56 days. Each diet was randomly allocated to three replicate tanks (*n* = 3). During the rearing period, the experimental conditions were described as follows: temperature: 22–32°C, total ammonia nitrogen <0.5 mg/L, dissolved oxygen level ≥6 mg/L, pH: 7.8–8.1, and nitrite <0.1 mg/L.

### 2.3. Sampling

After the 8-week feeding experiments, the prawns were starved for 24 hr prior to sampling. The prawns in each tank were counted and weighed to analyze the growth performance including survival, final body weight (FBW), weight gain (WG), and specific growth rate (SGR). The hepatopancreas weight of six prawns in each tank were measured to calculate the hepatopancreas index (HSI). The muscle weight of six prawns in each tank were recorded to calculate the dressing percentage. Total feed weight was used to determine the feed conversion rate (FCR). For the hemolymph collection, we prepared the anticoagulants according to the following formula: NaCl, 450 mM, KCl, 10 mM, EDTA · Na_2_, and 10 mM, HEPES (pH = 7.45), 10 mM. About 200 *μ*L of anticoagulant was added to each centrifuge tube and keeping anticoagulant: hemolymph at 1 : 1 (*v* : *v*). After thoroughly mixing the hemolymph and anticoagulant, the mixture was centrifuged at 6,000 *g* for 10 min. Finally, the hemolymph, muscle, and hepatopancreas were frozen in liquid nitrogen and retained at −80°C until use. The calculations are as follows:

Survival (%) = 100 × final shrimp number/initial shrimp number.

WG (%) = 100 × (final body weight−initial body weight)/ initial body weight.

SGR (%/day) = (Ln (final body weight)—Ln (initial body weight)) × 100/duration of experimental days.

HSI (%) = 100 × hepatopancreas wet weight/final body weight.

FCR = dry feed intake/body weight gain.

Dressing percentage (%) = 100 × muscle wet weight/final body weight.

### 2.4. Body Color and Astaxanthin Content Analysis

The skin color was measured with a method described previously by Han et al. [22]. In brief, prawns were packed in a bag and immersed at constant-temperature (80°C) water bath for 5 min. After cooling to room temperature, the surface moisture was wiped and the values of *L*^*∗*^, *a*^*∗*^, and *b*^*∗*^ were analyzed using a colorimeter (CS-210). *L*^*∗*^ values indicate lightness (range from 0 to 100); the *a*^*∗*^ values were used to assess the degree of redness or greenness. Negative values indicated a greener shade, while positive values indicated a redder shade; *b ^*∗*^* values indicated yellowness and blueness. Negative values indicated a bluer shade, while positive values indicated a yellower shade.

The astaxanthin contents of whole body, muscle, and hepatopancreas were measured as described before [23, 24]. In brief, dried samples were coarsely mixed with 5-mL ethyl acetate: ethanol (1 : 1 *v*/*v*) followed by centrifuging at 4,000 × *g* for 5 min. Once more, the precipitate was extracted using 5 mL of ethyl acetate and 5 mL of hexane till it became colorless. Three supernatants were combined, then dried with a nitrogen stream. After being resuspended in 1–2 mL of acetone containing 0.02% BHT, the samples were centrifuged for 5 min at 10,000 × *g*. The supernatant was measured by spectrophotometer (UV-2550, Shimadzu, Japan) at 474 nm to calculate astaxanthin contents.

### 2.5. Proximate Composition and Fatty Acid Profile Analyses

The levels of crude lipid, crude protein, and ash of feed, whole body and muscle were analyzed according to the Association of Official Analytical Chemists (AOAC; 1995). Crude protein was determined by the Kjeldahl method (*N* × 6.25, method 960.52; [25]) with a Kjeldahl apparatus (K355/K437, Buchi, Flawil, Switzerland). Following the sample's digestion with concentrated sulfuric acid, the boric acid solution and sodium hydroxide solution were added for distillation. The nitrogen content was calculated after titration with hydrochloric acid. Crude lipid content was measured according to the method 945.16 [25] by a Soxhlet apparatus (E816, Buchi, Flawil, Switzerland). The petroleum ether was continuously returned to extract for 12 hr under 50–55°C of water bath. Crude lipid content was measured by weighing the lipid packet after drying. Ash was measured with a burning method (method 942.05; [25]) in a muffle furnace (Beijing Kewei Instrument Co., Ltd., Beijing, China) at 550°C for 12 hr. The moisture was measured by freeze drying for 36 hr in a lyophilizer (Freeze Dryer LL1500, Thermo Fisher Scientific, Waltham, USA).

The analysis of fatty acid was conducted according to the method described before by Metcalfe et al. [26] with some modifications. In brief, 100 mg of ground sample was mixed with 3 mL potassium hydroxide–methanol solution and immersed at constant-temperature (72°C) water bath for 20 min. After cooling to room temperature, 3-mL hydrochloric acid–methanol solution was added to the mixture and immersed at constant-temperature (72°C) water bath for 20 min again. After cooling to room temperature, 1-mL hexyl hydride was added to the mixture and static at room temperature for 8 hr. The supernatant was centrifuged at 5,000 × *g* for 2 min and the fatty acid profiles were measured with a 7890B gas chromatograph (Agilent Technologies Inc., USA).

### 2.6. Hemolymph and Hepatopancreas Biochemical Parameters Analysis

The activities of glutamic pyruvic transaminase (GPT), glutamic oxaloacetic transaminase (GOT) in hemolymph were analyzed with the commercial kits (Nanjing Jiancheng Bioengineering Institute, China). In brief, before the formal measurement, the standard curve of GPT and GOT was made to ensure the *R*^2^ >0.99. After adding the matrix solution, dinitrophenylhydrazine, and sodium hydroxide solution in turn, the combination was measured using a microplate reader at 510 nm.

The activities of catalase (CAT) and superoxide dismutase (SOD), and malondialdehyde (MDA) and reduced glutathione (GSH) contents in hemolymph and hepatopancreas were measured by the commercial kits (Nanjing Jiancheng Bioengineering Institute). Hepatopancreas samples were homogenized using saline solution at a ratio of 1 : 9 (*W*/*V*) and hemolymph samples were diluted to a suitable concentration. The CAT activities and GSH contents were measured with a microplate reader at 405 nm. The MDA contents were measured with a microplate reader at 532 nm. The SOD activities were measured with a microplate reader at 450 nm.

### 2.7. Total RNA Extraction and Real-Time Quantitative PCR (RT-qPCR)

Total RNA from the hepatopancreas of *P*. *monodon* was extracted with TRIzol reagent (Takara, Japan) as described before by Zhao et al. [11]. The RNA quality and quantity were evaluated by a spectrophotometer (NanoDrop Technologies, Wilmington, USA). Immediately, the extracted RNA was then reverse transcribed with a HiScript III 1st Strand cDNA Synthesis Kit (Vazyme, China) in accordance with the manufacturer's protocol. The procedure of first step was 42°C for 5 min followed by the second step (37°C for 15 min and 85°C for 5 s). RT-qPCR was conducted using SYBR qPCR Master Mix (Vazyme) as per the established methods [27]. The procedure was incubated at 95°C for 3 min and then undergone 39 cycles. The procedure of cycle was 95°C for 10 s, 60°C for 10 s, and 72°C for 20 s. Based on the published sequences in NCBI, primers were created utilizing primer premier five software and the details are provided in Table 2. The housekeeping gene, *β-actin* was employed as a reference gene.

### 2.8. Transportation Stress Simulation Experiment

Six prawns from each tank were placed in a shake bed at 200 rpm with a low oxygen condition for 2 hr to simulate road transportation. At the end of the trial, *P*. *monodon* were anesthetized with MS-222, and the hemolymph and hepatopancreas were collected for the subsequent detection of antioxidant related indicators.

### 2.9. Statistical Analyses

All data presented in the study are presented as the mean ± standard deviation (SD). Polynomial contrasts analysis, including linear and quadratic patterns, was conducted using SPSS 23.0 software. Subsequently, Duncan's tests were employed to examine differences between the means. Statistical significance was defined as *P* < 0.05.

## 3. Results

### 3.1. Growth Performance

**Figure 1:**
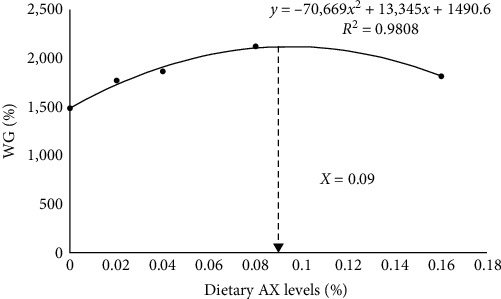
Quadratic regression analysis of weight gain (WG) against dietary graded leucine fed to juvenile *P. monodon* for 8 weeks.

The survival of *P*. *monodon* fed with different diets ranged from 80.00% to 92.00% and was independent of the dietary treatment (*P* > 0.05). With the rise of astaxanthin contents in diets, WG and SGR showed the trend of increasing first and then decreasing, and the maximum value appeared at AX0.08. The FCR of *P*. *monodon* showed significant linear pattern with the trend of decreasing first and then increasing, and the minimum value appeared at AX0.08. Dietary astaxanthin supplementation had no significant influence on HSI (*P* > 0.05). Compared with the control diet, AX 0.04 and AX0.16 significantly increased the dressing percent (*P* < 0.05; Table 3). Based on WG, the optimum requirement of dietary astaxanthin was estimated to be 0.09% of diet (90 mg kg^−1^) using second-order polynomial regression analysis (Figure 1).

### 3.2. Astaxanthin Content and Body Color Analysis

**Figure 2:**
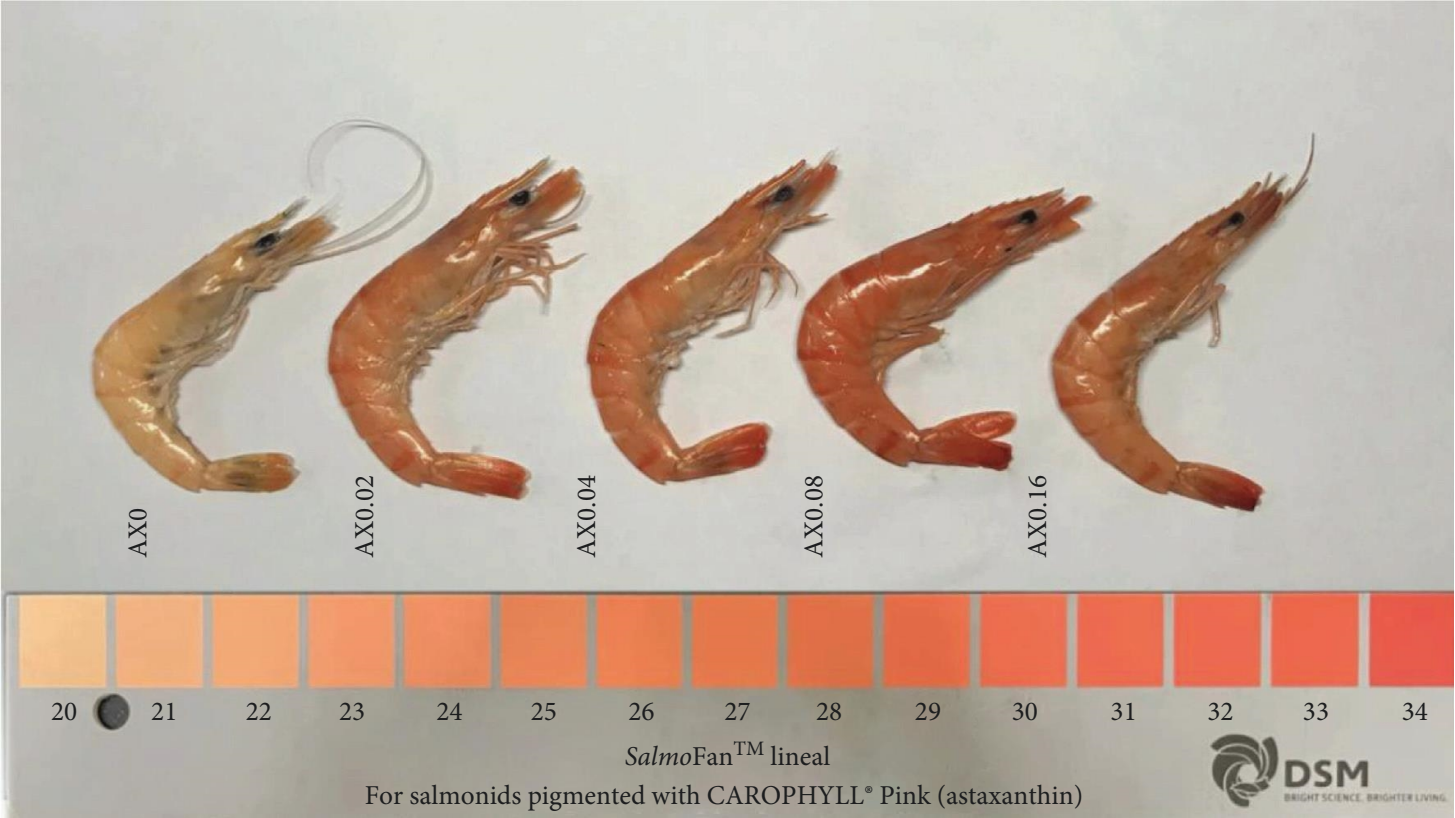
Color value of whole body in *P*. *monodon* fed different experimental diets.

Incremental levels of dietary astaxanthin significantly increased the astaxanthin deposition in the whole prawn body, muscle, and hepatopancreas (*P* < 0.05; Table 4). The astaxanthin deposition in the whole prawn body and hepatopancreas showed both significant linear and quadratic pattern with the dietary astaxanthin level, while linear pattern in muscle (Table 4). Compared with the SalmoFan card color, the highest value of SalmoFan card occurred in AX0.08 group (Figure 2). Additional study of color parameters in the carapace revealed a significant linear pattern in the values of lightness (*L*^*∗*^) with the rise in astaxanthin concentration in feed, showing a tendency of decreasing first and then increasing. The lowest *L*^*∗*^ value appeared at AX0.08. On the contrary, the values of redness (*a*^*∗*^) and yellowness (*b ^*∗*^*) showed a trend of increasing first and then decreasing, and the maximum value appeared at AX0.08 and AX0.04, respectively. The values of *a ^*∗*^* showed both significant linear and quadratic pattern with the dietary astaxanthin level. In the muscle, different doses of astaxanthin significantly reduced the *L*^*∗*^ values with a significant linear and quadratic pattern (*P* < 0.05). The trend of *a*^*∗*^ and *b*^*∗*^ values in muscle was similar with that in carapace (Table 5).

### 3.3. Proximate Compositions and Fatty Acid Profiles

According to Table 6, dietary astaxanthin had no significant influence in the moisture, crude protein, crude lipid, and ash contents in the whole prawn body and muscle (*P* > 0.05).

Dietary astaxanthin supplementation significantly influenced fatty acid profile in the whole prawn body and muscle. In the whole body, the levels of C16 : 0, C17 : 0, C18 : 0, and the whole saturated fatty acids (SFAs) were decreased by dietary astaxanthin with a significant linear pattern (*P* < 0.05). While astaxanthin increased the contents of C20 : 4n-6 and C20 : 5n-3 (eicosapentaenoic acid, EPA; Table 7). In muscle, dietary astaxanthin significantly decreased C16 : 0 levels with a linear and quadratic pattern, while increased the contents of EPA, C22 : 6n-3 (docosahexaenoic acid, DHA) and total n-3 PUFA with a significant linear pattern (*P* < 0.05). Compared with the control group, diet AX0.02 and AX0.08 decreased the contents of C18 : 1n-9, C18 : 1n-7, and C18 : 2n-6, while increased the levels of C20 : 1n-9, C20 : 4n-6, and total n-6 PUFA (*P* < 0.05). AX0.02 elevated the contents of C18 : 3n-6 and C18 : 3n-3 (*P* < 0.05; Table 8).

### 3.4. Antioxidation and Immune Parameters Analysis

**Figure 3:**
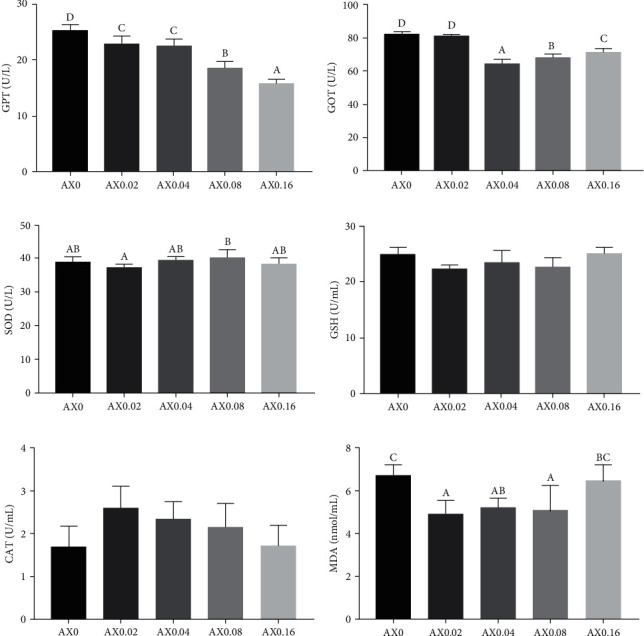
Effects of dietary astaxanthin supplementation on antioxidative indexes in hemolymph of *P*. *monodon* (*n* = 3). Bars bearing the same letters are not significantly different among treatments (*P* > 0.05), the same as below. (a) GPT: *P*_value_ ≤ 0.001, *P*_linear_ ≤ 0.001, *P*_quadratic_ = 0.053; (b) GOT: *P*_value_ ≤ 0.001, *P*_linear_ ≤ 0.001, *P*_quadratic_ ≤ 0.001; (c) SOD: *P*_value_ = 0.208, *P*_linear_ = 0.579, *P*_quadratic_ = 0.523; (d) GSH: *P*_value_ = 0.137, *P*_linear_ = 0.845, *P*_quadratic_ = 0.028; (e) CAT: *P*_value_ = 0.178, *P*_linear_ = 0.665, *P*_quadratic_ = 0.032; (f) MDA: *P*_value_ = 0.033, *P*_linear_ = 0.813, *P*_quadratic_ = 0.004.

**Figure 4:**
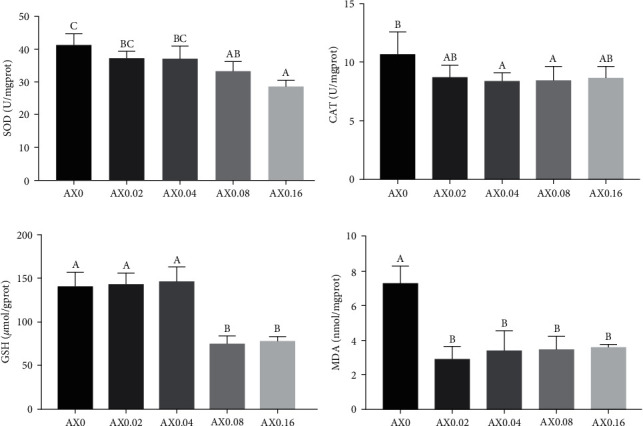
Effects of dietary astaxanthin supplementation on antioxidative indexes in hepatopancreas of *P*. *monodon* (*n* = 3). (a) SOD: *P*_value_ = 0.004, *P*_linear_ ≤ 0.001, *P*_quadratic_ = 0.495; (b) CAT: *P*_value_ = 0.143, *P*_linear_ = 0.059, *P*_quadratic_ = 0.086; (c) GSH: *P*_value_ ≤ 0.001, *P*_linear_ ≤ 0.001, *P*_quadratic_ = 0.024; (d) MDA: *P*_value_ ≤ 0.001, *P*_linear_ = 0.001, *P*_quadratic_ = 0.001.

Dietary astaxanthin supplementation had no influence on the activities of CAT, SOD and the contents of GSH in the hemolymph (*P* > 0.05). With the increase of astaxanthin level in feed, the activities of GPT in hemolymph were significantly decreased in a linear pattern (*P* < 0.05). The activities of GOT in hemolymph decreased initially, then increased with the increasing astaxanthin levels, but remained significantly lower than that of the control diet (*P* < 0.05). Compared with the control diet, AX0.02, AX0.04, and AX0.08 significantly decreased the contents of MDA (Figure 3). The effects of dietary astaxanthin on the antioxidation capability in hepatopancreas were stronger that in hemolymph. AX0.08 and AX0.16 significantly decreased the activities of SOD and the contents of GSH in hepatopancreas (*P* < 0.05). AX0.04 and AX0.08 decreased the activities of CAT (*P* < 0.05). Various doses of astaxanthin exhibited a significant reduction in MDA contents, demonstrating both linear and quadratic trends (*P* < 0.05; Figure 4).

**Figure 5:**
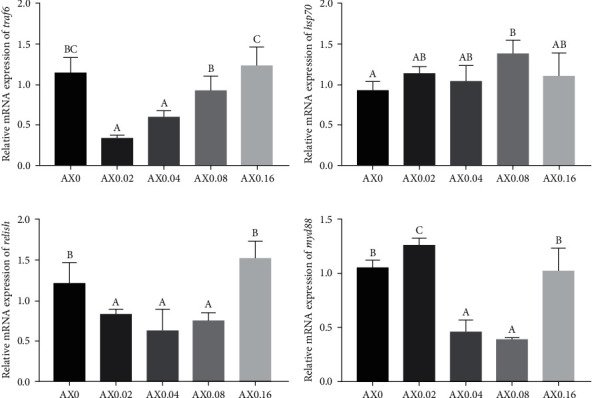
Effects of dietary astaxanthin supplementation on immune responses in hepatopancreas of *P*. *monodon* (*n* = 3). (a) *traf6*: *P*_value_ ≤ 0.001, *P*_linear_ = 0.022, *P*_quadratic_ ≤ 0.001; (b) *hsp70*: *P*_value_ = 0.088, *P*_linear_ = 0.082, *P*_quadratic_ = 0.184; (c) *relish*: *P*_value_ = 0.001, *P*_linear_ = 0.163, *P*_quadratic_ ≤ 0.001; (d) *myd88*: *P*_value_ ≤ 0.001, *P*_linear_ = 0.001, *P*_quadratic_ ≤ 0.001.

The mRNA expression of common immune genes in hepatopancreas was assessed to verify the effects of astaxanthin on nonspecific immunity of *P*. *monodon*. Compared with the control diet, the mRNA expression of *myd88*, *relish*, and *traf6* appeared a tendency of downregulated first and then upregulated with the increasing astaxanthin levels. Notably, AX0.08 significantly enhanced the mRNA expression of *hsp70* (Figure 5).

### 3.5. Resistance to Transportation Stress

**Figure 6:**
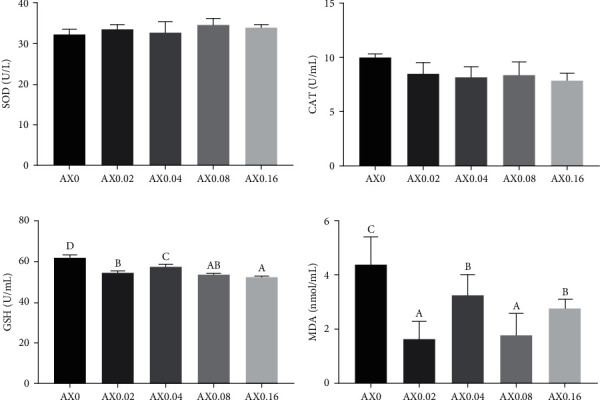
Effects of dietary astaxanthin supplementation on resistance to transportation stress of *P*. *monodon*. Antioxidative indexes in hemolymph were measured (*n* = 3). (a) SOD: *P*_value_ = 0.497, *P*_linear_ = 0.202, *P*_quadratic_ = 0.674; (b) CAT: *P*_value_ = 0.116, *P*_linear_ = 0.026, *P*_quadratic_ = 0.232; (c) GSH: *P*_value_ ≤ 0.001, *P*_linear_ ≤ 0.001, *P*_quadratic_ = 0.050; (d) MDA: *P*_value_ = 0.006, *P*_linear_ = 0.045, *P*_quadratic_ = 0.022.

**Figure 7:**
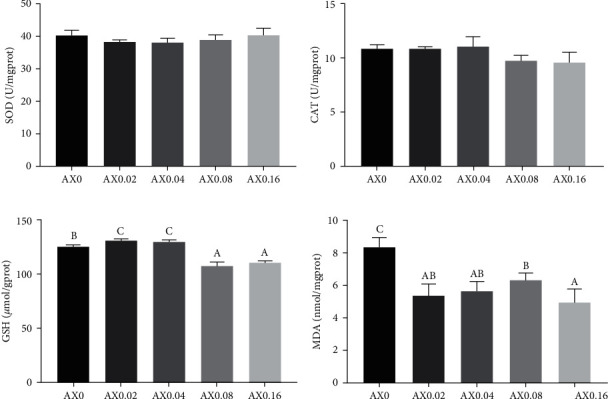
Effects of dietary astaxanthin supplementation on resistance to transportation stress of *P*. *monodon*. Antioxidative indexes in hepatopancreas were measured (*n* = 3). (a) SOD: *P*_value_ = 0.261, *P*_linear_ = 0.854, *P*_quadratic_ = 0.035; (b) CAT: *P*_value_ = 0.051, *P*_linear_ = 0.012, *P*_quadratic_ = 0.183; (c) GSH: *P*_value_ ≤ 0.001, *P*_linear_ ≤ 0.001, *P*_quadratic_ ≤ 0.001; (d) MDA: *P*_value_ = 0.001, *P*_linear_ = 0.001, *P*_quadratic_ = 0.029.

To investigate the effects of astaxanthin on enhancing the stress resistance of *P*. *monodon*, transportation simulation experiments were conducted on prawns at the end of feeding trial, and the antioxidant indexes in hemolymph and hepatopancreas were measured (Figures 6 and 7). Compare with the control diet, dietary astaxanthin supplementation had no influence on the activities of CAT and SOD in hemolymph and hepatopancreas. On the contrary, astaxanthin decreased the contents of GSH and MDA in hemolymph and hepatopancreas, indicating that astaxanthin enhancing the resistance of *P*. *monodon* to the transportation stress.

## 4. Discussion

The poor body color and weak stress resistance of *P. monodon* in the process of aquaculture forced people to find suitable feed additives. Numerous studies have demonstrated the positive effects of dietary astaxanthin in promoting growth performance [28], improving body color [29], and enhancing antioxidant capacity [30] in crustaceans. In the present study, different doses of dietary astaxanthin increased WGR and SGR compared with non-astaxanthin-supplemented shrimp. However, statistical analyses found that only the AX0.08 group had significant differences compared to the control diet. Lower or higher contents of astaxanthin did not significantly increase the growth performance of *P. monodon*, which was consistent with the results in red swamp crayfish (*Procambarus clarkii*) [31]. Further regression analysis showed that 0.09% astaxanthin (90 mg kg^−1^) meets the optimum nutrition demands of growing *P. monodon*. Previous studies indicated that astaxanthin intake of between 25 and 50 mg kg^−1^ is required for normal *P. monodon* growth and health [32]. However, Wade et al. [32] also demonstrated that 100 mg kg^−1^ astaxanthin still significantly increased the weight increment of *P. monodon*. The source and chemical structure of astaxanthin may be the reason for the above research differences. Overall, it is certain that appropriate dietary astaxanthin promotes the growth and health of *P. monodon*.

Body color is one of the important factors affecting muscle quality and consumption, which depends on absorption and deposition of pigment. Compared with natural feed, the uncertain availability of pigment in artificial feed causes abnormal skin color. Hence, how to accurately replicate and protect the natural luminous color of crustaceans is one of the biggest problems in aquaculture. Correspondingly, astaxanthin is widely used as feed additives to improve the body color of crustaceans. In the present study, dietary astaxanthin obviously regulated the values of color parameters, including *L*^*∗*^, *a*^*∗*^, and *b*^*∗*^. Similar results were appeared in red swamp crayfish [31], swimming crab (*Portunus trituberculatus*) [33], and whiteleg shrimp (*Litopenaeus vannamei*) [34]. In general, the pigmentation is closely related to dietary astaxanthin levels and the duration for which it is fed [35]. Overall, the present study revealed that dietary astaxanthin concentrations between 0.04% and 0.08% for 8 weeks were sufficient to produce optimal pigmentation in black tiger prawn. Maybe such a concentration of astaxanthin is just linked with the color protein crustacyanin in the hypodermal tissues to produce a bright color [36]. In addition, these results also demonstrated that crustacean tissues, particularly the carapace and muscle, have the ability to store massive amounts of astaxanthin. Hence, in order to make the color of prawns more favored by the consumers, it is necessary to add an appropriate amount of synthetic astaxanthin to the feed.

In addition to the role in pigmentation, astaxanthin also regulated the lipid metabolism. In mammals, studies showed that astaxanthin relieved lipid abnormal accumulation through inhibiting SREBP1 and activating PPAR-*α* [37]. Hence, nutritional enrichment with astaxanthin contributes to hepatic health in mammals. There are also many studies on astaxanthin regulating fatty acid profile in aquaculture [38, 39]. In general, astaxanthin increased the contents of n-3 PUFA and decreased total SFA levels, which was confirmed in the present study. Dietary astaxanthin significantly increased the EPA, DHA, and total n-3 PUFA levels in muscle and decreased the contents of C16 : 0 and total SFA. The muscle quality is closely related with the contents of n-3 PUFA. Hence, appropriate dietary astaxanthin can provide high-quality protein rich in n-3 PUFA for consumers. The above observations were also confirmed in *P. monodon* [32] and red porgy (*Pagrus pagrus*) [40]. However, study in tiger puffer (*Takifugu rubripes*) indicated that astaxanthin had no significant influence on the contents of EPA, DHA, and total n-3 PUFA [41]. The difference between species and sources may explain the differences in the above research. The positive function of astaxanthin in enhancing n-3 PUFA levels may rely on its antioxidant capacity, protecting n-3 PUFA from oxidation loss. In addition, the change of some fatty acids (C16 : 0, C18 : 0, C20 : 1n-9, etc.) in muscle is not a simple linear trend with the rise of astaxanthin, which was similar with the results in tiger puffer [41]. Previous study in Atlantic salmon (*Salmo salar*) indicated that the availability of dietary astaxanthin regulated the desaturation and elongation of fatty acids [42]. Hence, the decline of SFAs by astaxanthin may be due to the enhancement of desaturation and elongation processes, which also partially explains the increase in n-3 PUFA. However, the specific mechanism of astaxanthin regulating fatty acid metabolism still needs to be further explored.

Astaxanthin is among natural most potent antioxidants, activating the antioxidant system to eliminate extra reactive oxygen species *in vivo* [43]. In the present study, the antioxidant capacity of astaxanthin was reflected in two aspects. After the 8-week feeding trial, the lower GPT, GOT, SOD, CAT activities and MDA contents in treated *P. monodon* indicated improved antioxidant capacity and hepatopancreatic function. Compared with improving the growth performance and body color of aquatic animal, studies on astaxanthin improving antioxidant capacity are more concentrated in prawns and shrimp. Stressors include salinity stress [44], hypoxia stress [45], high-pH stress [46], and ammonia nitrogen stress [9]. In the present study, astaxanthin enhanced the resistance to hypoxia stress under transportation stress simulation by decreasing GSH and MDA contents. GSH is a critical antioxidant in the body. GSH mediates glutathione peroxidase (GPX) to exert antioxidant enzyme activity, catalyzing the conversion of H_2_O_2_ into H_2_O, and achieving a transition from reducing GSH to oxidizing glutathione (GSSG) during this process [47]. Hence, the decline in GSH content indicated the antioxidant system was activated by astaxanthin under transportation simulation experiment. However, some studies have also shown that astaxanthin increased the expression and activity of antioxidant enzymes, such as Chinese mitten crab (*Eriocheir sinensis*) [46, 48] and whiteleg shrimp [19]. The different trial environment, species, diets and stressors may explain these differences. In addition to enhance the antioxidant capacity, astaxanthin is also an important immune enhancer. Appropriate dietary astaxanthin decreased the mRNA expression of *traf6*, *myd88*, and *relish* in hepatopancreas of *P. monodon*. All the above genes can lead to the activation of NF-*κ*B and JUN, which are inflammation signal pathway. Hence, we speculate that astaxanthin enhanced prawn's immunity by decreasing the expression of the above genes and inhibiting inflammation. Excessive astaxanthin had the negative effects on the above gene expression. Similar results appeared at giant freshwater prawn (*Macrobrachium rosenbergii*), which indicated that excessive astaxanthin may have adverse effects on several enzymes involved in the immune system [49]. However, studies in whiteleg shrimp by Yu et al. [50] also indicated that astaxanthin increased the mRNA expression of immune-related genes, including *myd88*, *toll*, and *imd*, which demonstrated that astaxanthin enhanced the immunity. The upregulation or downregulation of immune gene expression by astaxanthin may be related to the aquaculture environment and species. Hence, further research is required to verify this mechanism of astaxanthin regulating immunity. Taken together, the function of astaxanthin on enhancing antioxidant capacity and nonspecific immunity improved the growth performance of *P. monodon*.

In conclusion, the results in the present study indicated that dietary astaxanthin had positive effects on the growth performance, pigmentation, antioxidant capacity, and nonspecific immunity of *P. monodon*. Based on WG, the optimum dietary astaxanthin requirement was 0.09% (90 mg kg^−1^). Regarding that *P. monodon* farming is plagued by various stressors and colorless, astaxanthin is an appropriate nutrition strategy to increase production and efficiency in the aquaculture.

## Figures and Tables

**Table 1:** Formulation and proximate composition of the basal diet (% of dry matter)^1^.

Ingredients (%)	Composition
Fish meal	20.00
Soybean meal	27.00
Chicken meal^a^	20.97
High-gluten flour^b^	22.59
Taurine^c^	0.40
Squid viscera meal	1.00
Fish oil^d^	1.00
Soybean oil^e^	0.80
Soybean lecithin	1.09
Cholesterol	0.80
Choline	0.80
Vitamin C	0.50
Monocalcium phosphate	1.00
Mineral mix^1^	1.00
Vitamin mix^2^	1.00
BHT	0.05
Proximate composition (dry matter %)	
Crude protein	46.85
Crude lipid	11.02
Ash	7.04
Gross energy (kJ/g)	33.92

^1^The basal diet was designed according to a published study in our lab [21]. ^a^Purchased from firm Tyson Foods Inc., USA. ^b^Purchased from Zhejiang Xinxin Biochemical Technology Co., Ltd., China. ^c^Purchased from Zhejiang NHU Co., Ltd., China. ^d^Purchased from Zhejiang Industrial Group Co., Ltd., China. ^e^ Purchased from Lijia Food Co., Ltd., China. ^1^Mineral premixes (g/kg mixture): KCl, 90 g; FeSO_4_ · 7H_2_O, 20 g; NaCl, 40 g; KI, 40 mg; CuSO_4_ · 5H_2_O, 3 g; ZnSO_4_ · 7H_2_O, 4 g; CaCO_3_, 215 g; CoSO_4_ · 7 H_2_O, 20 mg; MnSO_4_·H_2_O, 124 g; cellulose, 500 g. ^2^Vitamin premix, (g/kg mixture): VA, 2.5 g; VB_1_, 0.25 g; VB_2_, 1 g; VB_3_, 2.5 g; VB_5_, 5 g; VB_6_, 0.75 g; VB_7_, 2.5 g; VB_8_, 399 g; VB_9_, 0.25 g; VB_12_, 2.5 g; VD_3_, 6.25 g; VE, 7 g; VK_3_, 2.5 g; cellulose, 500 g.

**Table 2:** Primer sequences of genes used for cloning and qRT-PCR.

Primer	Sequences (5′–3′)	Accession number
*β-Actin* F	CACCGCCGAGCGAGAAATC	JQ241179
*β-Actin* R	CAGCGAGGAGGAGGAAGCA	
*myd88* F	AAGGGTGCCACAGATAGCAGAAC	KJ577578
*myd88* R	CACCTCTCCATGATGAGTTTAACAA	
*relish* F	AGGTGACAGAGGTGGGATGAGTT	JQ728539
*relish* R	CTCCAGTATTTGAATGAATGCCC	
*traf6* F	CGTGAGATCCTGCAGCTTAGTG	KJ577579
*traf6* R	GCACATGACTGGCTGAAAAGAA	
*hsp70* F	TCCTACGTCGCCTTCACAGAC	AF474375
*hsp70* R	CTTTGGCTTTGTGCTCTCGTT	

myd88, myeloid differentiation primary response protein 88; traf6, tnf receptor-associated factor 6; hsp70, heat shock 70 kDa protein.

**Table 3:** Growth performances of juvenile *P. monodon* fed different diets.

	Experiment diets							Polynomial contrasts	
	AX0	AX0.02	AX0.04	AX0.08	AX0.16	SEM	*P* value	Linear	Quad
Initial body wet weight (g)	0.28	0.27	0.27	0.27	0.30				
Final body wet weight (g)	4.43 ± 0.62	5.05 ± 0.75	5.31 ± 1.52	6.00 ± 0.29	5.74 ± 0.75	0.24	0.286	0.050	0.508
Weight gain rate (%)	1481.13 ± 221.16^a^	1769.56 ± 279.38^ab^	1865.98 ± 526.24^ab^	2122.32 ± 108.56^b^	1814.86 ± 248.61^ab^	89.10	0.268	0.113	0.169
Specific growth rate (%/d)	4.92 ± 0.26^a^	5.22 ± 0.27^ab^	5.27 ± 0.48^ab^	5.54 ± 0.09^b^	5.26 ± 0.23^ab^	0.08	0.229	0.089	0.168
Feed conversion rate	1.94 ± 0.49^a^	1.79 ± 0.21^ab^	1.68 ± 0.33^ab^	1.26 ± 0.20^b^	1.51 ± 0.20^ab^	0.09	0.147	0.032	0.484
Hepatopancreas index (%)	4.27 ± 0.23	4.28 ± 0.25	4.34 ± 0.18	4.27 ± 0.17	4.30 ± 0.06	0.04	0.986	0.873	0.794
Dressing percentage (%)	41.52 ± 0.41^a^	42.15 ± 1.13^ab^	47.20 ± 1.14^c^	41.85 ± 0.73^ab^	43.31 ± 0.96^b^	0.59	*P* ≤ 0.001	0.079	0.001
Survival (%)	86.67 ± 10.07	80.00 ± 10.58	84.00 ± 10.58	92.00 ± 8.00	84.00 ± 17.44	2.78	0.789	0.763	0.959

Means bearing the same superscript letters are not significantly different among treatments determined by Duncan's test (*P* > 0.05). Means ± standard deviation (SD); *n* = 3.

**Table 4:** Astaxanthin concentrations in different tissues of juvenile *P*. *monodon* fed different experimental diets (*μ*g/g).

	Experiment diets							Polynomial contrasts	
	AX0	AX0.02	AX0.04	AX0.08	AX0.16	SEM	*P* value	Linear	Quad
Whole body	6.63 ± 0.56^a^	15.21 ± 0.21^c^	12.84 ± 1.55^b^	16.92 ± 0.27^c^	16.82 ± 1.47^c^	1.05	*P* ≤ 0.001	*P* ≤ 0.001	0.001
Muscle	2.78 ± 0.38^a^	3.79 ± 1.65^ab^	4.91 ± 0.54^b^	4.45 ± 0.11^b^	4.96 ± 0.25^b^	0.28	0.038	0.007	0.169
Hepatopancreas	2.44 ± 0.27^a^	9.07 ± 0.39^b^	8.71 ± 1.34^b^	9.15 ± 0.28^b^	9.35 ± 0.96^b^	0.73	*P* ≤ 0.001	*P* ≤ 0.001	*P* ≤ 0.001

Means bearing the same superscript letters are not significantly different among treatments determined by Duncan's test (*P* > 0.05). Means ± SD; *n* = 3.

**Table 5:** Color parameters of juvenile *P*. *monodon* fed different experimental diets.

		Experiment diets							Polynomial contrasts	
		AX0	AX0.02	AX0.04	AX0.08	AX0.16	SEM	*P* value	Linear	Quad
Carapace	*L* ^*∗*^	64.70 ± 3.30^c^	61.68 ± 0.39^bc^	60.70 ± 1.06^b^	56.99 ± 0.91^a^	58.48 ± 1.49^ab^	0.81	0.003	*P* ≤ 0.001	0.125
	*a* ^*∗*^	5.01 ± 0.27^a^	15.15 ± 0.71^b^	17.76 ± 0.41^c^	24.23 ± 0.66^d^	16.67 ± 1.15^c^	1.67	*P* ≤ 0.001	*P* ≤ 0.001	*P* ≤ 0.001
	*b* ^*∗*^	23.73 ± 0.97^ab^	25.04 ± 0.21^bc^	26.94 ± 1.00^c^	26.00 ± 0.83^c^	22.48 ± 1.79^a^	0.49	0.004	0.458	*P* ≤ 0.001

Muscle	*L* ^*∗*^	73.58 ± 1.28^d^	65.45 ± 0.68^bc^	66.24 ± 2.58^c^	63.10 ± 0.67^b^	55.67 ± 1.23^a^	1.54	*P* ≤ 0.001	*P* ≤ 0.001	*P* ≤ 0.001
	*a* ^*∗*^	4.32 ± 0.86^a^	10.28 ± 0.56^b^	13.90 ± 1.81^c^	17.14 ± 0.14^d^	13.74 ± 0.35^c^	1.13	*P* ≤ 0.001	*P* ≤ 0.001	*P* ≤ 0.001
	*b* ^*∗*^	20.19 ± 1.96^a^	21.59 ± 0.45^ab^	22.64 ± 0.75^b^	21.23 ± 0.31^ab^	20.12 ± 0.45^a^	0.32	0.026	0.767	0.003

Means bearing the same superscript letters are not significantly different among treatments determined by Duncan's test (*P* > 0.05). Means ± SD; *n* = 3.

**Table 6:** Body and muscle proximate composition in juvenile *P*. *monodon* fed different experimental diets.

	Experiment diets							Polynomial contrasts	
	AX0	AX0.02	AX0.04	AX0.08	AX0.16	SEM	*P* value	Linear	Quad
Whole body									
Moisture (%)	64.93 ± 0.99	66.42 ± 2.63	66.96 ± 1.04	65.63 ± 0.70	66.01 ± 1.88	0.40	0.616	0.652	0.268
Protein (%)	71.66 ± 2.92	73.74 ± 1.19	74.05 ± 0.24	72.81 ± 1.27	73.02 ± 0.55	0.40	0.416	0.541	0.146
Lipid (%)	4.58 ± 0.15	4.39 ± 0.56	4.84 ± 0.58	4.60 ± 0.67	4.80 ± 1.07	0.15	0.924	0.612	0.952
Ash (%)	13.69 ± 1.01	14.22 ± 1.07	13.96 ± 1.11	14.44 ± 0.18	13.38 ± 0.77	0.22	0.632	0.808	0.236
Muscle									
Moisture (%)	69.37 ± 3.90	72.03 ± 1.51	70.35 ± 0.98	72.24 ± 0.65	72.53 ± 1.19	0.55	0.305	0.107	0.795
Protein (%)	90.22 ± 0.41	90.84 ± 1.11	91.87 ± 1.41	89.90 ± 1.28	89.98 ± 2.83	0.40	0.568	0.640	0.270
Lipid (%)	3.48 ± 1.16	3.27 ± 0.94	2.87 ± 0.12	2.94 ± 0.29	2.22 ± 0.49	0.19	0.324	0.056	0.734
Ash (%)	6.47 ± 0.65	5.92 ± 0.25	5.99 ± 0.05	5.89 ± 0.31	6.15 ± 0.45	0.10	0.414	0.384	0.119

(Means ± SD; *n* = 3).

**Table 7:** Fatty acid profile (% total fatty acids) in the whole body of *P*. *monodon* fed different experimental diets ^*∗*^.

	Experiment diets							Polynomial contrasts	
	AX0	AX0.02	AX0.04	AX0.08	AX0.16	SEM	*P* value	Linear	Quad
C14 : 0	1.57 ± 0.60	1.40 ± 0.70	1.86 ± 0.75	0.82 ± 0.21	1.04 ± 0.45	0.62	0.268	0.153	0.578
C16 : 0	24.37 ± 0.09^b^	21.43 ± 1.00^a^	22.01 ± 0.61^a^	21.83 ± 0.12^a^	21.60 ± 0.83^a^	1.24	0.001	0.001	0.008
C16 : 1n-7	2.07 ± 0.41	1.85 ± 0.20	1.83 ± 0.41	2.15 ± 0.15	1.85 ± 0.23	0.29	0.585	0.812	0.763
C17 : 0	1.85 ± 0.78^bc^	2.30 ± 0.38^c^	2.21 ± 0.73^c^	0.68 ± 0.13^a^	1.11 ± 0.35^ab^	0.8	0.015	0.010	0.227
C18 : 0	13.46 ± 0.37^c^	11.05 ± 1.41^ab^	12.33 ± 1.17^bc^	9.52 ± 0.25^a^	9.35 ± 1.48^a^	1.88	0.003	0.001	0.874
C18 : 1n-9	15.54 ± 1.99	17.51 ± 0.77	15.99 ± 3.40	20.33 ± 0.45	18.67 ± 2.34	2.89	0.241	0.091	0.812
C18 : 1n-7	2.30 ± 0.03	2.35 ± 0.89	2.08 ± 0.15	2.34 ± 0.02	2.82 ± 0.59	0.48	0.493	0.288	0.213
C18 : 2n-6	13.30 ± 2.74^a^	15.72 ± 2.53^ab^	15.03 ± 2.86^ab^	18.50 ± 0.17^b^	16.28 ± 1.30^ab^	2.55	0.134	0.053	0.300
C18 : 3n-6	2.26 ± 0.88	2.09 ± 1.31	2.28 ± 0.65	1.34 ± 0.11	1.76 ± 0.33	0.75	0.564	0.246	0.975
C18 : 3n-3	1.55 ± 0.58	1.63 ± 0.78	1.59 ± 0.52	1.12 ± 0.14	1.47 ± 0.35	0.12	0.763	0.497	0.931
C20 : 1n-9	1.78 ± 0.95	1.80 ± 0.27	2.01 ± 0.62	1.69 ± 0.07	1.46 ± 0.37	0.13	0.805	0.473	0.400
C20 : 4n-6	5.80 ± 0.15^ab^	6.94 ± 3.94^ab^	7.66 ± 0.18^ab^	4.01 ± 0.88^a^	8.46 ± 1.52^b^	0.59	0.117	0.512	0.604
C20 : 5n-3	2.51 ± 0.30^a^	3.17 ± 1.12^ab^	1.96 ± 0.75^a^	5.38 ± 0.29^c^	3.73 ± 0.18^b^	0.34	0.001	0.003	0.994
C22 : 6n-3	10.50 ± 2.78	10.75 ± 1.80	10.98 ± 1.24	9.30 ± 0.08	9.74 ± 1.52	0.41	0.730	0.365	0.689
SFA	41.24 ± 1.00^c^	36.18 ± 2.90^ab^	38.40 ± 2.13^bc^	32.85 ± 0.49^a^	33.10 ± 2.07^a^	0.95	0.001	*P* ≤ 0.001	0.509
MUFA	21.70 ± 0.81	23.51 ± 4.35	21.91 ± 3.25	26.50 ± 0.67	24.79 ± 3.25	3.06	0.278	0.110	0.894
n-3 PUFA	14.56 ± 1.90	15.55 ± 0.35	14.53 ± 0.10	15.80 ± 0.36	14.94 ± 2.01	1.19	0.649	0.671	0.612
n-6 PUFA	21.36 ± 1.80^a^	24.75 ± 3.04^ab^	24.97 ± 2.26^ab^	23.84 ± 0.86^ab^	26.50 ± 1.25^b^	2.43	0.092	0.028	0.525
n-3/n-6	0.68 ± 0.03^c^	0.63 ± 0.07^abc^	0.58 ± 0.05^ab^	0.66 ± 0.01^bc^	0.56 ± 0.06^a^	0.02	0.058	0.041	0.859

SFA, saturated fatty acid; C14 : 0, C16 : 0, C17 : 0, C18 : 0. MUFA, monounsaturated fatty acids; 16 : 1n–7, 18 : 1n-7, 18 : 1n-9, 20 : 1n-9. n-3 PUFA: 18 : 3n-3, 20 : 5n-3, 22 : 6n-3. n-6 PUFA: C18 : 2n-6, C18 : 3n-6, C20 : 4n-6. ^*∗*^The contents of some fatty acids were too minor to detected, such as C20 : 0, C22 : 0, C24 : 0, C14 : 1, C22 : 1 n-11, C20 : 2 n-6, C20 : 3 n-6, C18 : 4 n-3, C22 : 5 n-3, are not listed in the table. Means bearing the same superscript letters are not significantly different among treatments determined by Duncan's test (*P* > 0.05). Means ± SD; *n* = 3.

**Table 8:** Fatty acid profile (% total fatty acids) in the muscle of *P*. *monodon* fed different experimental diets ^*∗*^.

	Experiment diets							Polynomial contrasts	
	AX0	AX0.02	AX0.04	AX0.08	AX0.16	SEM	*P* value	Linear	Quad
C14 : 0	1.27 ± 0.40	1.50 ± 0.20	1.52 ± 0.39	1.25 ± 0.18	1.31 ± 0.31	0.07	0.720	0.769	0.366
C16 : 0	27.02 ± 2.16^c^	18.08 ± 1.67^a^	22.13 ± 0.92^b^	16.48 ± 0.35^a^	22.06 ± 0.30^b^	1.02	*P* ≤ 0.001	0.001	*P* ≤ 0.001
C16 : 1n-7	1.16 ± 0.27	1.41 ± 0.20	1.13 ± 0.32	1.49 ± 0.41	1.08 ± 0.28	0.08	0.404	0.888	0.331
C17 : 0	1.30 ± 0.21	1.26 ± 0.22	1.29 ± 0.52	1.11 ± 0.06	1.16 ± 0.41	0.07	0.927	0.479	0.967
C18 : 0	10.37 ± 1.82^a^	11.20 ± 1.32^ab^	13.48 ± 0.24^c^	9.92 ± 0.55^a^	13.04 ± 0.77^bc^	0.45	0.008	0.070	0.602
C18 : 1n-9	10.87 ± 1.02^b^	7.59 ± 0.70^a^	10.91 ± 0.89^b^	8.68 ± 0.13^a^	11.12 ± 0.72^b^	0.42	*P* ≤ 0.001	0.278	0.005
C18 : 1n-7	1.68 ± 0.21^bc^	1.35 ± 0.12^a^	1.54 ± 0.22^ab^	1.31 ± 0.11^a^	1.85 ± 0.10^c^	0.06	0.011	0.324	0.004
C18 : 2n-6	11.56 ± 1.32^b^	8.38 ± 0.33^a^	11.11 ± 0.20^b^	9.59 ± 0.13^a^	11.61 ± 0.58^b^	0.37	*P* ≤ 0.001	0.311	0.002
C18 : 3n-6	1.55 ± 0.43^a^	2.33 ± 0.34^b^	1.37 ± 0.09^a^	1.62 ± 0.35^a^	1.56 ± 0.44^a^	0.12	0.056	0.310	0.536
C18 : 3n-3	1.37 ± 0.26^a^	2.23 ± 0.32^b^	1.27 ± 0.20^a^	1.17 ± 0.02^a^	1.34 ± 0.29^a^	0.11	0.002	0.030	0.355
C20 : 1n-9	1.61 ± 0.03^a^	3.32 ± 1.12^b^	1.72 ± 0.41^a^	3.41 ± 0.79^b^	2.75 ± 0.35^ab^	0.25	0.017	0.077	0.325
C20 : 4n-6	19.25 ± 1.92^b^	27.41 ± 0.82^c^	19.07 ± 0.45^b^	26.31 ± 1.00^c^	14.33 ± 0.32^a^	1.33	*P* ≤ 0.001	*P* ≤ 0.001	*P* ≤ 0.001
C20 : 5n-3	3.50 ± 0.34^a^	5.20 ± 0.79^b^	5.59 ± 0.55^b^	7.92 ± 0.76^c^	5.81 ± 0.53^b^	0.40	*P* ≤ 0.001	*P* ≤ 0.001	0.002
C22 : 6n-3	6.37 ± 0.30^a^	8.26 ± 1.23^ab^	7.85 ± 1.64^ab^	9.26 ± 0.31^bc^	10.97 ± 0.84^c^	0.46	0.003	*P* ≤ 0.001	0.520
SFA	39.96 ± 0.57^c^	32.04 ± 2.85^b^	38.42 ± 1.03^c^	28.77 ± 1.11^a^	37.57 ± 0.91^c^	1.18	*P* ≤ 0.001	0.016	*P* ≤ 0.001
MUFA	15.32 ± 1.04^ab^	13.66 ± 1.34^a^	15.30 ± 0.72^ab^	14.89 ± 0.37^a^	16.79 ± 0.62^b^	0.33	0.020	0.028	0.025
n-3 PUFA	11.25 ± 0.14^a^	15.68 ± 1.59^b^	14.70 ± 1.48^b^	18.36 ± 0.50^c^	18.12 ± 1.11^c^	0.74	*P* ≤ 0.001	*P* ≤ 0.001	0.079
n-6 PUFA	32.36 ± 3.22^b^	38.11 ± 1.34^c^	31.56 ± 0.20^b^	37.51 ± 1.27^c^	27.49 ± 0.42^a^	1.12	*P* ≤ 0.001	0.007	*P* ≤ 0.001
n-3/n-6	0.35 ± 0.04^a^	0.41 ± 0.05^ab^	0.47 ± 0.04^bc^	0.49 ± 0.03^c^	0.66 ± 0.03^d^	0.03	*P* ≤ 0.001	*P* ≤ 0.001	0.054

^*∗*^The contents of some fatty acids were too minor to detected, such as C20 : 0, C22 : 0, C24 : 0, C14 : 1, C22 : 1 n-11, C20 : 2 n-6, C20 : 3 n-6, C18 : 4 n-3, C22 : 5 n-3, are not listed in the table. Means bearing the same superscript letters are not significantly different among treatments determined by Duncan's test (*P* > 0.05). Means ± SD; *n* = 3.

## Data Availability

The data used to support the findings of this study are included within the article.

## Additional Points

*Highlights*. According to the second-order polynomial regression analysis based on the weight gain, the optimum requirement of dietary synthetic astaxanthin was estimated to be 0.09% of diet (90 mg kg^−1^) for *P. monodon*. Appropriate dietary astaxanthin improved body color, enhanced the antioxidant capacity and nonspecific immunity of *P. monodon*. Dietary astaxanthin supplementation increased the contents of n-3 PUFA in muscle of *P. monodon*.
